# The molecular basis of defective lens development in the Iberian mole

**DOI:** 10.1186/1741-7007-6-44

**Published:** 2008-10-21

**Authors:** F David Carmona, Rafael Jiménez, J Martin Collinson

**Affiliations:** 1 School of Medical Sciences, Institute of Medical Sciences, University of Aberdeen, Foresterhill, Aberdeen, AB25 2ZD, UK; 2 Departamento de Genética e Instituto de Biotecnología, Facultad de Ciencias, Universidad de Granada, 18071 Granada, Spain

## Abstract

**Background:**

Fossorial mammals face natural selection pressures that differ from those acting on surface dwelling animals, and these may lead to reduced visual system development. We have studied eye development in a species of true mole, the Iberian mole *Talpa occidentalis*, and present the molecular basis of abnormal lens development. This is the first embryological developmental study of the eyes of any fossorial mammal at the molecular level.

**Results:**

Lens fibre differentiation is not completed in the Iberian mole. Although eye development starts normally (similar to other model species), defects are seen after closure of the lens vesicle. *PAX6 *is not down-regulated in developing lens fibre nuclei, as it is in other species, and there is ectopic expression of *FOXE3*, a putative downstream effector of *PAX6*, in some, but not all lens fibres. FOXE3-positive lens fibres continue to proliferate within the posterior compartment of the embryonic lens, but unlike in the mouse, no proliferation was detected anywhere in the postnatal mole lens. The undifferentiated status of the anterior epithelial cells was compromised, and most of them undergo apoptosis. Furthermore, β-crystallin and *PROX1 *expression patterns are abnormal and our data suggest that genes encoding β-crystallins are not directly regulated by PAX6, c-MAF and PROX1 in the Iberian mole, as they are in other model vertebrates.

**Conclusion:**

In other model vertebrates, genetic pathways controlling lens development robustly compartmentalise the lens into a simple, undifferentiated, proliferative anterior epithelium, and quiescent, anuclear, terminally differentiated posterior lens fibres. These pathways are not as robust in the mole, and lead to loss of the anterior epithelial phenotype and only partial differentiation of the lens fibres, which continue to express 'epithelial' genes. Paradigms of genetic regulatory networks developed in other vertebrates appear not to hold true for the Iberian mole.

## Background

Reduced visual systems are common among vertebrates adapted to live in subterranean habitats [1]. Studies on blind cave-fish (*Astyanax mexicanus*) have provided valuable insights about evolutionary eye development. This species has become a useful model for studying the molecular biology of eye organogenesis in vertebrates. The eyes of the blind cave-fish begin to develop relatively normal but there is a loss of *pax6 *expression in the lens secondary to increased midline expression of the morphogen sonic hedgehog (*shh*), leading to apoptosis and degeneration of other eye structures [2,3]. In mammals, the marsupial moles (*Notoryctes *spp.) represent the most extreme case described. These animals exhibit vestigial closed eyes without lenses [4]. Grant's golden moles (*Eremitalpa granti*) and blind mole rats (*Spalax ehrenbergi*) also show very small eyes completely covered by skin, although a rudimentary lens with disorganised fibre cells, that have not extruded their nuclei, is present [5,6]. Perhaps surprisingly, the latter species expresses crystallin genes in its undifferentiated lenses [7]. The naked mole rats (*Heterocephalus glaber*) have eyelids, but they are generally closed unless the animals are alarmed. The lens of these subterranean rodents seems to float freely inside the eyeball and exhibits various irregularities in shape [8]. The European moles (*Talpa europaea*) have less degenerated eyes in which the main eye structures are present; however, the lens shows disorganised nucleated lens fibres [9]. In spite of this, these immature fibre cells express genes encoding α-, β- and γ-crystallins, markers typically specific to differentiated lens fibres [7]. Although it seems that the lens of European moles has lost its function in vision, these animals are capable of discriminating between dark and light stimuli [10].

Very few molecular studies have been performed in fossorial mammals [6-8,11]. For instance, nothing is known about the genetic control of eye morphogenesis in the true moles (Talpidae), but it is likely that they represent a good model of the first steps of evolutionary eye degeneration [7,9].

Lens differentiation is a key event in eye development. It has an important role in the formation of the anterior segment of the eye and loss of the lens leads to failure of anterior eye structures [12,13]. Normal lens development requires precise regulation of gene expression and cell proliferation, because the epithelial precursors of the anterior side remain undifferentiated, whereas those of the posterior part begin to differentiate as primary lens fibres, which elongate towards the anterior surface occluding the lens vesicle. Subsequent secondary lens fibres are added from a narrow zone of the anterior epithelium located in the equator of the lens, the germinative zone, due to the continuous differentiation of epithelial cells situated in this region [14-16]. In later stages, mitotic activity is restricted almost exclusively in the germinative zone [17-19].

Differentiating lens fibre cells elongate, lose their nuclei and organelles (including the Golgi apparatus, endoplasmic reticulum and mitochondria) and synthesise specific soluble proteins involved in lens function: α-, β- and γ-crystallins [17,19,20]. Fibre cell denucleation comprises an enzymatic mechanism similar to those of apoptosis, requiring the caspase family of proteases pathway [20,21].

Many genes involved in lens development have been identified, including *PAX6*/*Pax6*, which is highly conserved across metazoans [22-24]. *PAX6 *expression in lens development is dynamic. This gene is highly expressed in the lens placode [25,26]. *PAX6 *expression is normally maintained in proliferating cells of the anterior lens epithelium, and it is down-regulated in posterior lens fibres to enable terminal differentiation [25-28]. PAX6 regulates the expression of a wide range of genes, including crystallins that are required for light transparency, refraction and maintenance of lens integrity [29,30]. On one hand, it is a positive transcriptional activator of genes encoding different lens epithelial cells specific crystallins, like αA-, αB-, δ1- and ζ-crystallin [31-35]. On the other hand, PAX6 is a repressor of the differentiated lens fibre markers β-crystallins [27,28,36].

*FOXE3/Foxe3 *encodes a transcription factor essential for lens epithelial proliferation and closure of the lens vesicle, and is regulated by Pax6 in mice [37,38]. It has been proposed that many of the ocular malformations associated with *PAX6 *haplo-insufficiency are consequences of a reduced expression of *FOXE3 *[38].

In this paper we have studied the whole process of lens differentiation in the Iberian mole (*Talpa occidentalis*, Cabrera 1907), in order to determine whether the eye defects of these subterranean insectivores are a consequence of a degenerated or a developmentally retarded visual system. Our results show that the development of the lens is not completed in the Iberian mole and that changes in the regulation of both *PAX6 *and *FOXE3 *expression underlie the eye dysmorphology of this species. Furthermore, our data suggest that expression of β-crystallin genes is not directly repressed by PAX6 in the mole, unlike in other model species.

## Results

### Morphology of the eye and lens of the Iberian mole

**Figure 1:**
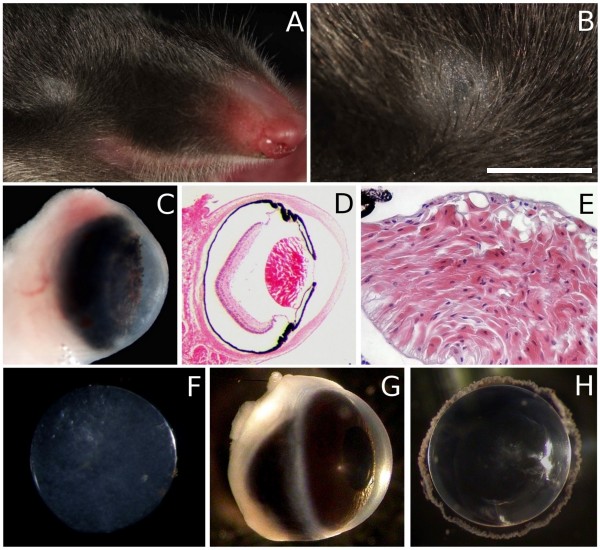
**Morphological characteristics of the eye of the Iberian mole**. The eyes of the Iberian mole are largely hidden in the fur (A) and permanently closed due to the absence of eyelids (B). The cornea is transparent and the iris has numerous bulges (C). Haematoxylin and eosin staining of wax sections showed that the eye had the main structures and looked functional (D) but undeveloped due to the presence of nuclei in the disorganised lens fibres (E). The mole lens exhibited some transparency and had no acute cataracts (F). Wild-type mouse eye (G) and lens (H) are shown as a model of normal mammalian eye phenotype. Scale bar represents 7 mm in A, 1.5 mm in B, 800 μm in C and D, 170 μm in E, 400 μm in F, and 2 mm in G and H.

Morphological analysis showed that Iberian moles have small eyes with permanently closed eyelids completely covered by skin (Figure 1A and 1B). Behind a relatively transparent cornea, there is a pigmented iris with numerous bulges (Figure 1C), unlike that of the wild-type mouse (*N *> 20; see Figure 1G). No muscle tissue was detected in the iris, and the posterior edge of the iris adheres to the lens capsule (Figure 1D). Haematoxylin and eosin staining showed that the corneal epithelium is one or two cell layers thick, in contrast to the 5–8 layers observed in humans and mice (Figure 1D). The retina, which seems to have a relatively normal layered structure, is small and does not extend to the edges of the globe (Figure 1D). The lens is composed of disorganised and nucleated lens fibres (Figure 1E). It is biconvex and has no acute cataracts (Figure 1F), although it is not as transparent as the wild-type mouse lens (see Figure 1H).

**Figure 2:**
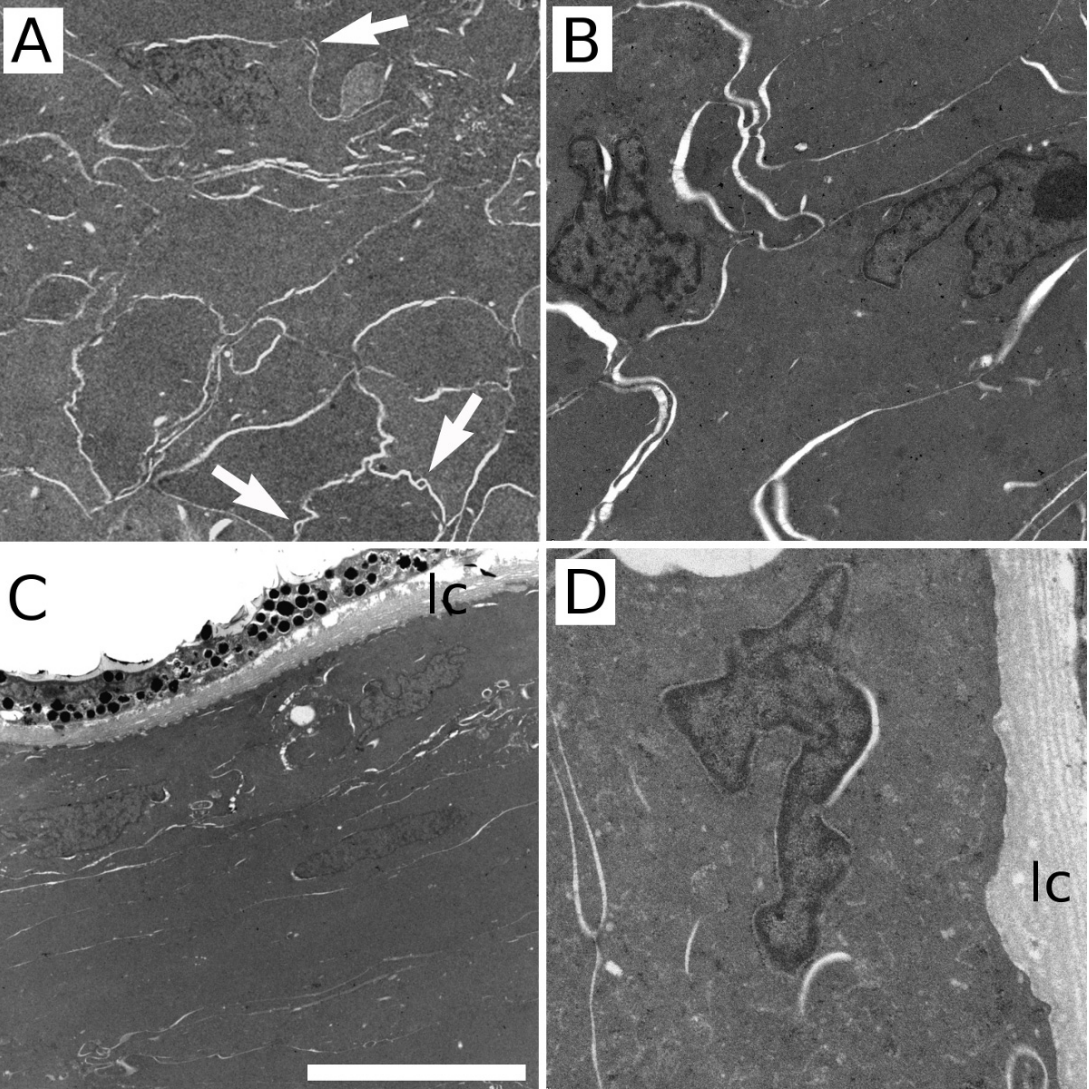
**Transmission electron micrographs of the Iberian mole adult lens**. Lens fibres appeared clearly disorganised (A), although some 'ball-and-socket' connections were observed between them (arrows). The nuclei of the fibre cells showed irregular shapes and no mitochondria were observed in the cytoplasm (B). The lens epithelium was composed of flattened cells (C) and, as in the lens fibres, no mitochondria were seen in any epithelial cell at higher magnification (D). Mole lens was covered by a typical lens capsule (lc). Scale bar represents 10 μm in A, 4 μm in B, 12 μm in C and 2 μm in D.

Transmission electron microscopy (TEM) showed that the lens is surrounded by a lens capsule and there are normal 'ball and socket' connections between lens fibres, although they are scarce (Figure 2). The lens fibre cells have irregular nuclei (in contrast to anuclear fibre cells in other mammals) and lack obvious mitochondria (Figure 2B). The lens epithelium is not composed of cuboidal cells as in other mammals, but of flattened cells morphologically and ultrastructurally quite similar to the observed nucleated lens fibres (Figure 2C). No basal lamina was observed in all the samples analysed. Similarly, no mitochondria were seen in the cytoplasm of the epithelial cells (Figure 2D).

**Figure 3:**
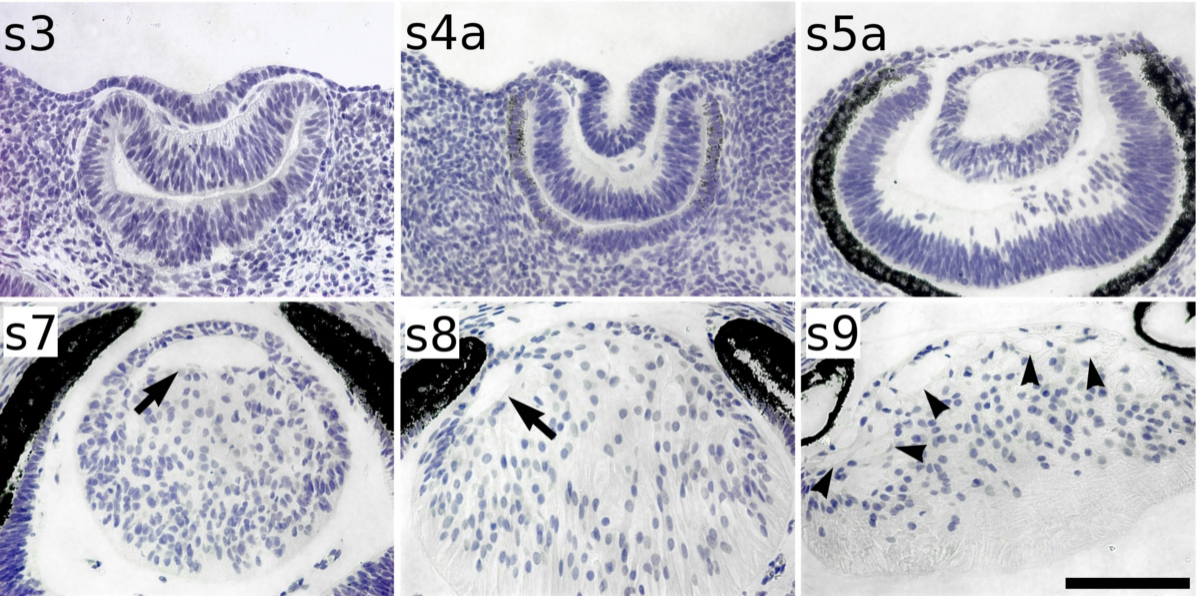
**Haematoxylin staining on mole wax sections**. In the Iberian mole, the lens placode is established at the end of the s3 stage, that is, about 13 days post-coitum (dpc) and it rapidly invaginates forming the lens pit (s4a, 14 dpc). At s5a (17 dpc), the lens is clearly polarised and the posterior cells have already begun to elongate. About 5 days later (s7, about 22 dpc), lens fibre cells have not reached the centre of the lens epithelium (arrow). Lens occlusion has not been completed at the last prenatal stage (s8, about 26 dpc), as a small lens cavity is still observed (arrow). After birth, several vacuoles in the epithelium/fibre cells boundary (arrowheads) are seen in the lens of newborn moles (s9, 1 day post-partum), a situation maintained until adulthood (not shown). Scale bar represents 100 μm in all figures.

Haematoxylin staining of tissue sections showed that the mole lens begins to develop properly. The lens vesicle detaches completely from the overlying surface ectoderm and become polarised (Figure 3). However, important differences were found in relation to mouse lenses at similar stages. One difference is that a conspicuous lens cavity is observed until the last prenatal stage, that is, s8, 24–28 days post-coitum (dpc). This means that lens occlusion lasts around 10 days. Primary lens fibre elongation is very slow in moles compared with mice, in which this process only takes 2 days (from 11.5 to 13.5 dpc; [39]). Furthermore, several vacuoles appeared in the epithelium-lens fibres borderline at the s9 stage (newborn moles) and remained there even in adulthood, unlike mouse lenses.

The analysis suggested that although the mole lens was transparent, there was some failure of normal developmental processes, with incomplete differentiation of both lens epithelium and lens fibres. A molecular analysis was therefore performed.

### *PAX6 *is not down-regulated throughout lens fibre differentiation in moles

**Figure 4:**
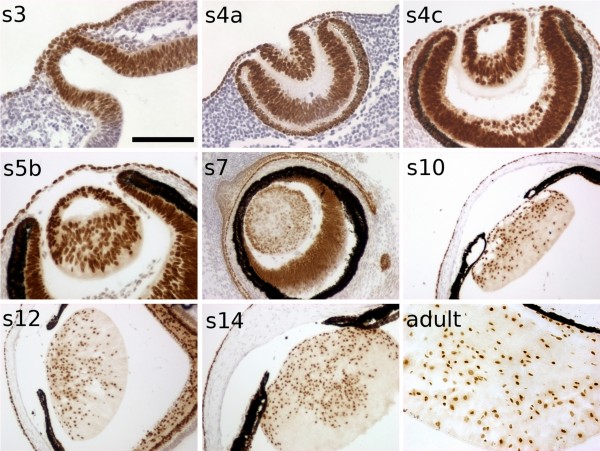
***PAX6 *expression pattern during lens development in the Iberian mole**. *PAX6 *is expressed during the whole development of the mole lens. PAX6 protein is detected in both the head ectoderm of the presumptive lens and the optic vesicle at the s3 stage, as well as in the optic cup and invaginating lens placode at the next stage (s4a). From the onset of primary lens fibre differentiation (s4c) until adulthood, *PAX6 *is expressed in all the cells forming the lens, including both the anterior epithelial cells and nucleated lens fibres. Scale bar represents 200 μm in s10, s12 and s14 figures, and 100 μm in all the rest.

By immunohistochemistry, PAX6 was detected in the cornea, iris, lens, retina and their precursors during the whole eye development in the Iberian mole (Figure 4), as expected. At the s3 stage (equivalent to E9 in mice), *PAX6 *was expressed in the optic vesicle as well as in the adjacent surface ectoderm. The optic cup and lens vesicle were strongly positive for PAX6 at the next stage (s4a, E10 in mice). From stage s4c (E11 in mice) on, the posterior cells begin to elongate. At later stages, primary lens fibre elongation continues until stage s8 (equivalent to mouse E18), in which the lens cavity is almost closed. In mice, expression of *Pax6 *is maintained in the lens epithelium, but not in lens fibres: differentiation of lens fibre cells has been shown to require the down-regulation of *Pax6 *[28]. In contrast, in the mole, PAX6 was detected in all lens nuclei, including those located in the posterior region throughout development. The nuclei of the lens fibres are not extruded at any point and remain PAX6-positive during the whole life of the animal.

### *FOXE3 *is expressed in some differentiating lens fibre cells during mole embryogenesis

**Figure 5:**
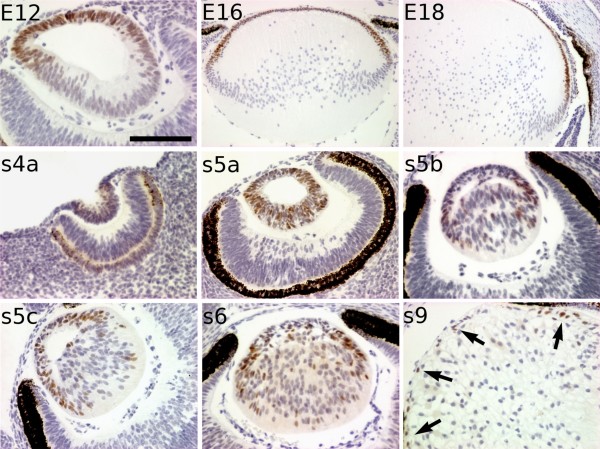
**Anomalous profile of *FOXE3 *expression in the lens of the Iberian mole**. In the mouse lens, *Foxe3 *expression becomes polarised as soon as the primary lens fibres begin to differentiate (E12), remaining only in the epithelial cells but not in the differentiating lens fibres. This pattern is maintained in later stages (E16, E18) until adulthood. During mole lens development, FOXE3 is weakly detected in the lens pit (s4a stage). At the equivalent stage of mouse E12 (s5a), most of the cells, but not all, show a high expression of *FOXE3*, including the elongating primary lens fibres. In subsequent stages (s5b-s6), FOXE3 is observed mainly in the nuclei of cells located in the germinative zone, but also in single cells in both sides of the lens. At the s9 stage (newborn moles), *FOXE3 *is only expressed in a few epithelial cells (arrows). No FOXE3 was detected during the rest of developmental stages (not shown). Scale bar represents 200 μm in E16 and E18 figures, and 100 μm in all the rest.

We hypothesised that retention of *PAX6 *expression in lens fibre nuclei was causal to a failure of full differentiation, and tested this by examining the expression of another lens epithelial gene, *FOXE3*. In the mouse lenses, *Foxe3 *expression is similar to that of *Pax6*, with early expression throughout the developing lens pit becoming restricted to lens epithelial cells only during development [37]. In the mole, *FOXE3 *is weakly expressed in the invaginating lens placode at s4a stage (Figure 5). Its expression increased after the establishment of the lens vesicle reaching a peak at s5a. At this point, most of the lens cells were strongly positive for FOXE3. However, some cells appeared less immunoreactive in both the anterior and posterior regions of the lens. This pattern differs from that observed in the equivalent stage in mice (E12), in which all the epithelial cells expressed *Foxe3*, whereas presumptive lens fibres became Foxe3-negative. In subsequent stages (s5b-s6), this transcription factor was detected mainly in the germinative zone, although there were also individual positive cells in all part of the lens. We confirmed that *Foxe3 *is expressed in all the epithelial cells but not in the lens fibres at equivalent stages in mouse samples. In the s9 stage of mole development, some (but not all) cells of the lens epithelium exhibited FOXE3-specific immunoreactivity. FOXE3 was not detected during the rest of the mole lens development. As not all the lens fibre cells that expressed ectopic *PAX6 *also expressed ectopic *FOXE3*, the data suggested that the dysgenic phenotype of the mole lens was not solely due to *PAX6*-induced misregulation of *FOXE3*. The close link between *Pax6 *and *Foxe3 *expression found in the mouse was lost in the mole, suggesting that there were further adaptations of the genetic pathways controlling eye development.

### *PROX1 *and *c-MAF *expressions pattern during mole lens development

**Figure 6:**
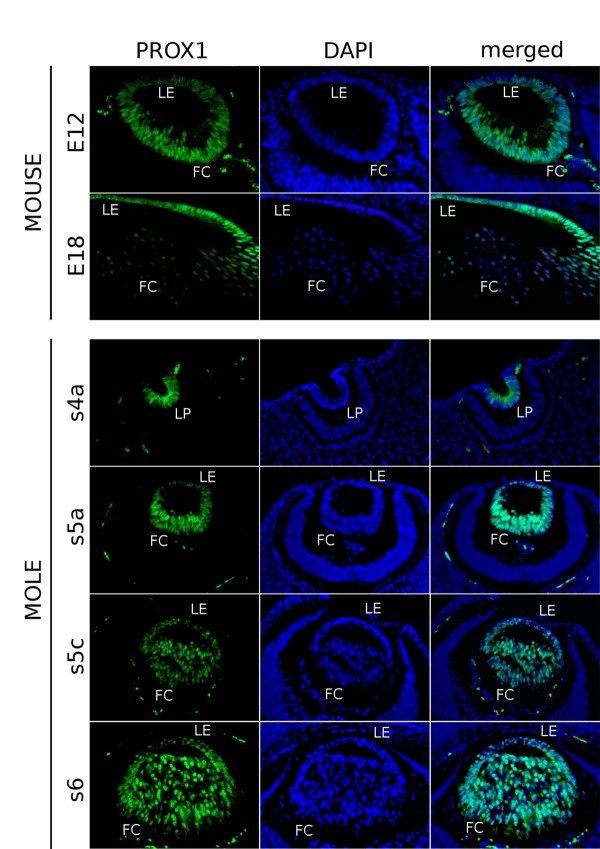
**Spatiotemporal expression pattern of *PROX1 *in the Iberian mole lens**. In mouse samples (E12, E18), specific Prox1-immunofluorescence is observed in the nucleus of differentiating lens fibres and in the cytoplasm of epithelial cells. During the mole lens development, PROX1 showed a cytoplasmic distribution in the invaginating lens placode (s4a). Once the lens vesicle becomes polarised, PROX1 is highly detected in the nucleus of all the lens cells (s5a, s5c). From the s6 stage on, cytoplasmic localisation of PROX1 is clearly seen mainly in posterior fibre cells but also in the lens epithelium. Photomicrographs were taken using a single bandpass fluorescence mirror unit and merged with 'The Gimp' software. Scale bar represents 100 μm in all figures. LE, lens epithelium; FC, fibre cells; LP, lens pit.

PROX1 and c-MAF play important roles in lens fibre differentiation [16]. In the mouse lens, Prox1 is primarily detected in the nuclei of the differentiating lens fibres but in the cytoplasm of the lens epithelial cells (mouse rows in Figure 6; [40]). The spatiotemporal expression pattern of *PROX1 *in the lens of the Iberian mole clearly differs from that observed in mouse. In the mole, PROX1 was detected in the cytoplasm of the cells forming the invaginating lens placode at the s3 stage (Figure 6). During primary lens fibre differentiation, both epithelial and fibre cell nuclei expressed *PROX1*. A clear increase in cytoplasmic PROX1-immunoreactivity was observed from stage s6 (mainly in the lens fibres), although the main signal was in the nuclei (Figure 6, last row). This weaker cytoplasmic expression was maintained until adulthood. In postnatal stages, including the adult, some fibre cell nuclei appeared less PROX1-immunoreactive (not shown), and the fluorescence was barely detectable in some nuclei.

**Figure 7:**
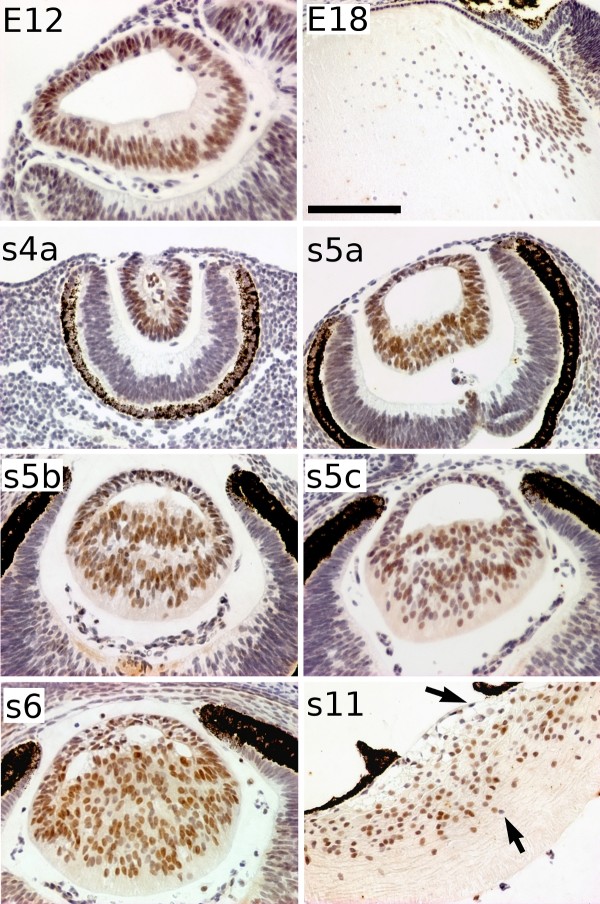
**Immunodetection of c-MAF in different stages of the Iberian mole lens development**. At 12 dpc (E12), *c-Maf *is expressed in all the cells of the mouse lens. At later stages (E18), it is down-regulated in the epithelial cells, being expressed mainly in the differentiating lens fibres. In the mole, c-MAF is detected in the lens pit (s4a). From the beginning of primary lens fibre differentiation, fibre cells appeared more immunoreactive than epithelial cells, although both cell types were clearly c-MAF-positive (s5a-s6). However, some single cells showed no evident *c-MAF *expression in postnatal stages (s11) (arrows). Scale bar represents 200 μm in E18 figure, and 100 μm in all the rest.

Expression of *c-MAF*, which encodes a transcription factor required for lens fibre differentiation, was relatively normal in the mole lens. c-MAF protein was detected in all lens cells during the first stages of lens development (until mole s5a, equivalent to mouse E12) in both moles and mice (Figure 7). From stage s5b (E13 in the mouse), high c-MAF-immunoreactivity was observed in the differentiating lens fibres of the moles, as in the mouse. In contrast to the mouse, where *c-Maf *expression is barely detectable in the lens epithelium at later stages (E18 in Figure 7; [41,42]), the protein was clearly present in the epithelia in equivalent stages of the mole, although still at lower levels than in the lens fibres. Some cells appeared c-MAF-negative during mole postnatal stages: most of them were located in the anterior epithelium (arrows in Figure 7).

### Abnormal cell proliferation pattern during mole lens development

**Figure 8:**
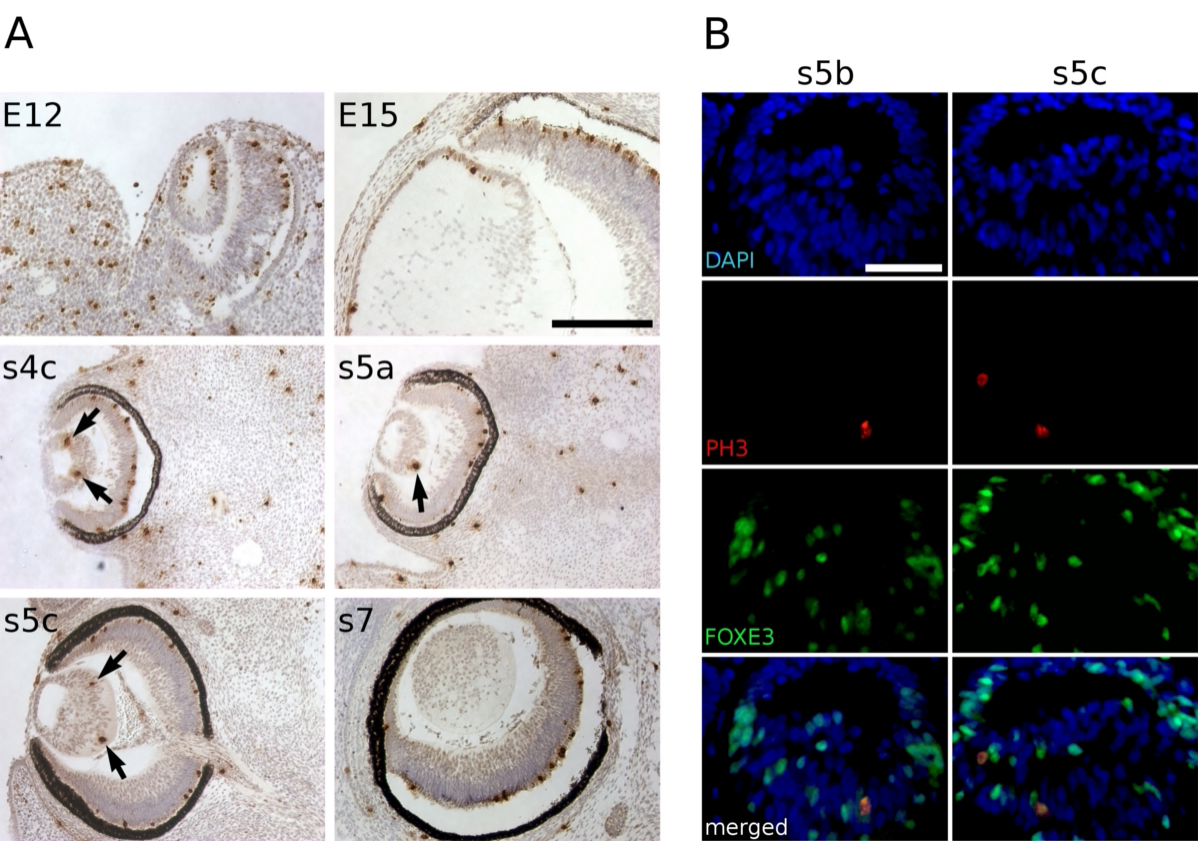
**Lens fibre cells proliferation in the Iberian mole**. (A) 3,3'-Diaminobenzidine tetrahydrochloride-immunostaining for PH3 on both mouse and mole wax sections at different embryonic stages. During mouse lens differentiation, proliferating cells (immunoreactive for PH3) are always found in the anterior epithelium (E12, E15). In the mole, very few PH3-positive cells were detected between the s4c and s5c stages, and most of them were located in the posterior region of the lens (arrows). No proliferation was observed in the lens at s7 and subsequent stages. (B) Double-immunofluorescence for PH3 (red) and FOXE3 (green) on wax sections of s5b and s5c mole samples. All the PH3-positive cells showed immunoreactivity against the anti-FOXE3 antibody. The nuclei are stained blue with 4'6-diamidino-2-phenylindole stain. Photomicrographs were taken using a single bandpass fluorescence mirror unit and merged with 'The Gimp' software. Black scale bar represents 200 μm in all figures of A, and white scale bar represents 50 μm in all figures of B.

Cell proliferation was studied by analysing the presence of the phosphorylated form of the histone H3 (PH3). In wild-type mouse samples, this protein was observed in some cells throughout the lens vesicle at E12 (Figure 8A), but was restricted to the lens epithelium by E15, as described previously [18]. In the mole, PH3-positive cells were scarce in comparison to equivalent stages in mice. From stage s4c on, only one or two mitotic lens cells were seen per section, with most sections showing no proliferating cells. PH3 was frequently detected in the elongating cells located at the posterior side of the lens between stages s5a and s6. No proliferating cells were observed at s7 and later stages of the mole lens development (*N *≥ 4 lenses/stage), whereas PH3 labelling was observed in all mouse lens epithelia at all stages. Hence patterns of proliferation are spatially deregulated in the mole lens, and proliferation is prematurely terminated with lens growth after s7 presumably relying solely on the increasing size of lens fibres.

Consistent with the apparent role of FOXE3 in maintaining proliferation of lens cells, double immunostaining for PH3 and FOXE3 showed that all PH3-positive cells were also expressing *FOXE3 *(*N *≥ 5 lenses) (Figure 8B). This suggests that ectopic expression of *FOXE3 *in some lens fibres could be involved in the abnormal proliferation of lens fibre cells observed in the Iberian mole.

### Epithelial cells undergo apoptosis in the mole lens

**Figure 9:**
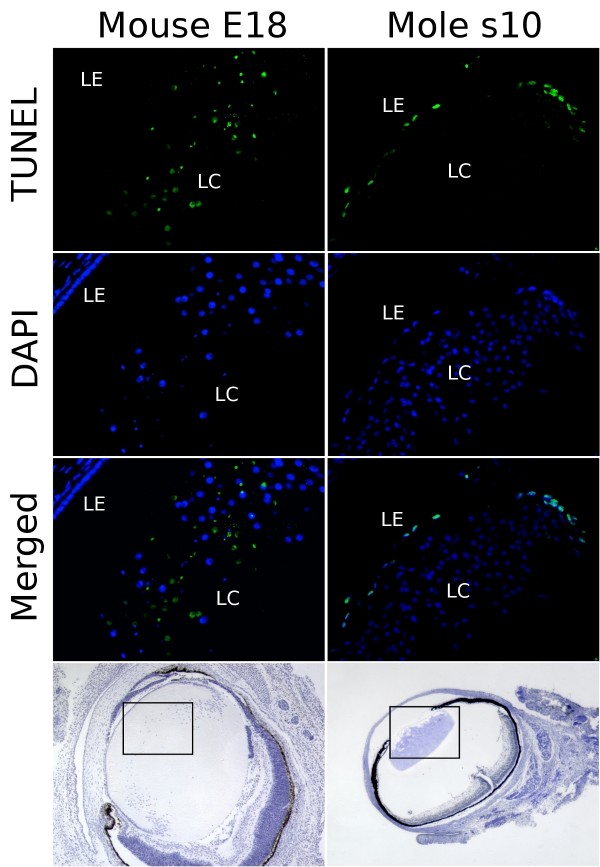
**TUNEL assay in mouse and mole lenses**. Terminal deoxynucleotidyl transferase (TdT)-mediated dUTP nick-end labelling (TUNEL)-positive cells are observed in the lens core (LC) but not in the lens epithelium (LE) of 18 days post-coitum mice embryos (E18). In contrast, TUNEL reaction was detected in most of the epithelial cells but not in the lens fibres of infant moles from the s10 stage. The nuclei are stained blue with 4'6-diamidino-2-phenylindole stain. Haematoxylin photomicrographs from the same sections used in TUNEL analysis are shown in the bottom row at lower magnifications to assess lens structure. Scale bar represents 100 μm in the immunofluorescence photomicrographs and 400 μm in the haematoxylin-stained sections.

Immunohistochemical data, combined with the morphological analysis of lens abnormalities above, suggested that the robust genetic pathways, which distinguish the lens epithelial and lens fibre cells, are beginning to break down in the Iberian mole, with epithelial cells exhibiting some aspects of the fibre cell phenotype, and vice versa. This hypothesis was tested further. Apoptotic-like pathways have been described during lens fibre differentiation in mice, but no apoptosis is normally observed in the wild-type lens epithelium [20]. This observation was confirmed in our observations of E18 wild-type mouse lenses, using the terminal deoxynucleotidyl transferase (TdT)-mediated dUTP nick-end labelling (TUNEL) reaction (see Methods). DNA double-breaks take place in the elongated mouse fibre cells as a consequence of the apoptotic processes that lead to the loss of their nuclei (see Figure 9, left column). A reverse situation was seen in the mole, where no evidence of apoptosis was found in the lens at any embryonic stage. However, DNA double-breaks were detected in the anterior epithelium of infant moles from stage s10 (the second postnatal stage) (Figure 9, right column). Almost all epithelial cells, including those located in the germinative zone, appeared strongly or weakly TUNEL-positive, whereas no signal was observed in any lens fibre (*N *≥ 6 lenses). This situation is the opposite of that observed in E18 mouse embryos. The apparent apoptotic event is restricted to the immediate postnatal stages, because the TUNEL reaction was negative in all the mole adult lenses examined (*N *≥ 3). The abnormal apoptosis profile observed in the lens epithelium of infant moles is very similar to that described in *Foxe3*-mutant mice [37]. Therefore, the down-regulation of *FOXE3 *in many lens epithelial cells could be responsible for the apparent loss of the fully differentiated epithelial cell type phenotype in the mole.

### β-crystallins are present in PAX6-positive cells in the mole lens

**Figure 10:**
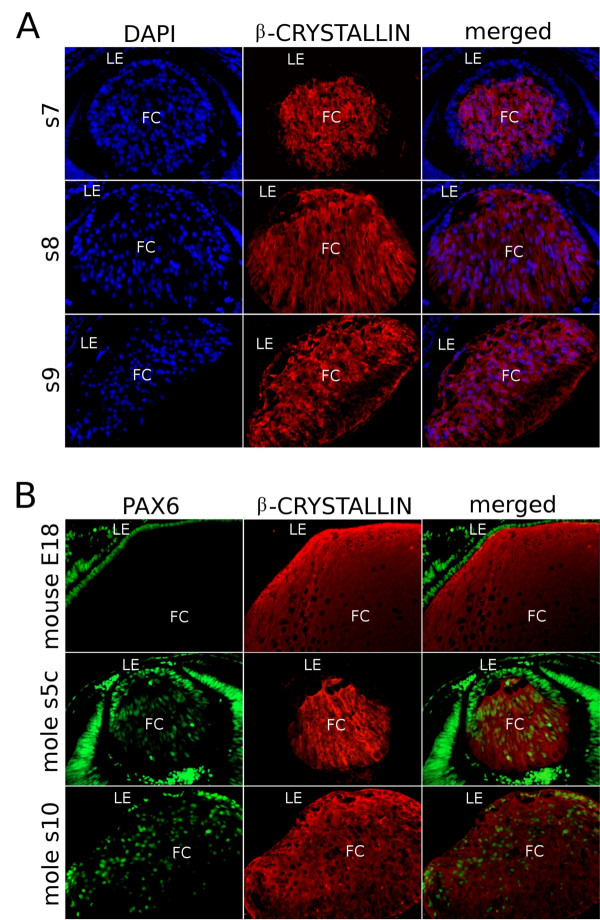
**β-crystallin genes are expressed in PAX6-positive cells, including epithelial cells, in the lens of the Iberian mole**. (A) The lens epithelium remains β-crystallin-negative at the s7 stage, but a weak β-crystallin expression is observed at the s8 stage. From s9 to adulthood, both fibre and epithelial cells show a similar β-crystallin-immunofluorescence. (B) In E18 mice samples, β-crystallin (red) is detected in the cytoplasm of lens fibres and Pax6 (green) in the nucleus of epithelial cells. In s5c mole embryos, the lens epithelium appeared PAX6-positive and β-crystallin-negative, but the fibre cells were positive for both proteins. At s10, all the lens cells, including the epithelial cells, showed immunoreactivity against both anti-PAX6 and anti-β-crystallin antibodies. The nuclei are stained blue with 4'6-diamidino-2-phenylindole stain. Photomicrographs were taken using a single bandpass fluorescence mirror unit and merged with 'The Gimp' software. Scale bar represents 100 μm in all figures of both A and B. LE, lens epithelium; FC, fibre cells.

The function of fibre cells in mole lenses was investigated using β-crystallin as a marker for terminal differentiation. In mice and chickens, β-crystallin genes are expressed only in lens fibre cells that have down-regulated *Pax6*, but not in epithelial cells where *Pax6 *expression is maintained. Pax6 is reported to be a transcriptional repressor of genes encoding β-crystallins [27,28,36]. In the mouse, using an antibody that recognises all β-crystallin isoforms, we confirmed that expression of *Pax6 *and β-crystallin is mutually exclusive – lens fibres were β-crystallin-positive and Pax6-negative, whereas all lens epithelial cells were Pax6-positive and β-crystallin-negative. During mole lens development, β-crystallins were first detected at the s5c stage (Figure 10), but not at previous stages (not shown). These proteins were located in the cytoplasm of the elongating posterior cells: the lens epithelium was β-crystallin-negative until stage s8. Hence, at stages s5c-s7, when the first defects of molecular patterning of the lens were being noted (the altered patterns of *PAX6*, *FOXE3 *and *PROX1 *expression described above), the expression of β-crystallins was normal, with expression in lens fibre cells but not the epithelium. Nevertheless, weak immunoreactivity against the anti-β-crystallin antibody was observed later, in epithelial cells at the last prenatal stage (s8) (Figure 10A), and similar high levels of β-crystallin expression were detected in both the epithelial cells and the lens fibres from the s9 stage on (lower rows in Figure 10A and 10B). Figure 10B shows that, in contrast to the mutually exclusive patterns of expression observed in wild-type mouse lenses, all the cells expressing β-crystallins are PAX6-positive. Only those cells located in the lens epithelium between stages s5c and s7, are immunoreactive for PAX6 but not for β-crystallins (*N *≥ 4 lenses/stage). These data suggest a breakdown of the inhibitory effect of *PAX6 *expression on lens fibre differentiation, consistent with the results above.

## Discussion

### Moles as models of a dysgenic lens phenotype

Our results show that the eyes of the Iberian mole, although permanently closed, are morphologically relatively normal and we do not believe that this species is totally blind. The eyelids of the Iberian mole are thin and translucent (FDC and JMC, unpublished observations), and their eyes have an apparently functional anterior segment with only minor overt morphological abnormalities of the cornea, such as the reduced thickness of the corneal epithelium, suggesting that the barrier function of this tissue may be redundant. The pupil diameter is probably fixed due to the absence of iris musculature, but the retina is well developed and our pilot data (not shown) indicate that the optic nerve successfully projects to the visual cortex. Molecular analyses of the developing retina and cornea will be presented elsewhere (FDC and JMC, manuscripts in preparation). However, it is the lens that shows the most overt abnormal phenotype, and, given the known role of the vertebrate lens as an organising centre for eye development, it is plausible that the other anterior segment defects are to some extent secondary to the failure of paracrine signalling from the lens to anterior eye tissues [43,44].

Iberian moles are closely related to the European mole, which has open eyes, with potentially better visual acuity [45,46]. Current knowledge on eye degeneration and disease are based mainly on the study of mutant mice or transgenic mice in which targeted mutations have been induced [47]. Fossorial mammals represent additional models of eye degeneration, since these species have evolved from surface-dwelling ancestors whose visual acuity may not have been very different from the modern-day mouse. In moles, molecular pathways involved in eye development have undergone changes leading to a wide range of eye degeneration phenotypes [1]. It would be predicted that it might be possible to reconstruct stages in evolutionary eye degeneration by studying the molecular genetics of ocular development across a phylogenetic reconstruction of the sighted and non-sighted talpids.

The data presented here show that the abnormal lens in Iberian moles represents a series of primary developmental defects and is not solely an adult degenerative condition. Fibre cell differentiation and maturation in mouse and human lenses is associated with denucleation. In the mouse, the denucleation process is largely completed by postnatal day 1, so that there are no nucleated fibres in the centre of the lens at birth [48]. Our results from TEM showed that lens fibres were disorganised and nucleated in the adult mole. This immature feature is in contrast with the fact that fibre differentiation is at least partly complete, that is, no mitochondria were observed in mole lens fibres, as in mouse, and some, but not many, mature 'ball and socket' connections between fibres were present. Hence, it seems clear that lens fibre differentiation is triggered in the mole lens, but it is not concluded. Adult lens epithelial cells in the mole did not show the cuboidal structure characteristic of mouse lenses, and also lacked mitochondria, indicating that the normal epithelial phenotype observed in other mammals is lost during mole lens development – mole epithelial cells showed a partial lens fibre phenotype. This phenotype is consistent with the possibility that lens polarity is disrupted by failure of the signalling mechanisms controlling the proliferation/differentiation switch in the lens epithelium.

### Ectopic *PAX6 *expression leads to defective lens development in the Iberian mole

PAX6 plays a crucial role in lens development in all vertebrate eyes [23,49]. In most species, once the lens becomes polarised *PAX6/Pax6 *expression is restricted exclusively to the lens epithelium, and alterations in this profile lead to severe lens anomalies [25,26]. Transgenic mice in which *Pax6 *is ectopically expressed in lens fibres, exhibit abnormal fibre cell elongation and incomplete denucleation [28]. Their lenses showed cataracts and a lumen between the apical surfaces of the epithelial and fibre cells. This ectopic *Pax6 *expression in fibre cells induces a reduction in the protein levels of c-Maf and β-crystallins, indicating that *Pax6 *inactivation is necessary for lens fibre differentiation and maturation [28]. Similarly, overexpression of *Pax6 *in the mouse also causes cataracts, with partial failure of lens fibre differentiation and abnormalities in fibre shape as well as fibre cell/lens capsule and fibre cell/fibre cell interactions [50,51].

Loss of *pax6 *expression precedes lens apoptosis and subsequent eye degeneration in the blind cave-fish [2]. In contrast, the expression of this gene is maintained throughout eye development in the Iberian mole. Adult lenses of the mole, in which PAX6 is present in lens fibres, showed similar morphological characteristics than those of transgenic mice ectopically expressing *Pax6 *in fibre cells (abnormal fibre cell elongation, impaired denucleation and vacuoles in the border with the lens epithelium). However, no cataracts were observed in any of the mole lenses analysed, probably due to a higher β-crystallin levels in the lens fibres, in comparison to the overexpressing transgenic mice. In the 'PAX77' transgenic mouse line, which overexpresses human *PAX6 *from a YAC transgene, occasional pockets of PAX6-positive cells are seen within the lens fibre region, and some of these can be labelled with bromodeoxyuridine, indicating ectopic proliferation [51,52]. Our results show that proliferation is not inhibited in the posterior region of the mole lens, even after lens fibres begin to elongate, consistent with the maintenance of *PAX6 *expression in this species. Nevertheless, the proliferation rate in the eye of the Iberian mole is very low in relation to the mouse, which may be the cause of its reduced size.

### Impaired expression of *FOXE3 *could cause loss of undifferentiated status and apoptosis in the lens epithelium of the Iberian mole

Lens epithelial cells are flattened and lack apparent mitochondria in the adult mole, and TUNEL labelling showed that DNA double-breaks were present in most lens epithelial cells in infant moles, with no apoptotic cell detected in the posterior region of the lens. The data suggest that lens epithelial cells are lost during development in the mole and that the surviving lens anterior cells are fibre-like. TUNEL-positive cells were first detected, just when the vacuoles between the lens epithelium and the lens fibres are formed and correlated with loss of FOXE3, a winged helix-forkhead transcription factor. This abnormal apoptotic profile seems not to be conserved in other fossorial mammals with naturally reduced eyes, like the naked mole rat, in which TUNEL reaction is not detected in the lens epithelium at any postnatal stage [8].

*Foxe3 *is thought to be activated by Pax6 at around 9 dpc in the mouse lens placode. This gene remains active in all the cells of the lens vesicle until 12.5 dpc, when it is down-regulated in the elongating primary lens fibres [37,38]. *Foxe3 *continues to be expressed in the mouse lens epithelium until adulthood, being indispensable for a complete closure of the lens vesicle and for the maintenance, proliferation and survival of the lens epithelial cells [37]. *Foxe3 *is very sensitive to Pax6 dosage, and it has been proposed that many of the ocular malformations associated with *Pax6 *haplo-insufficiency are consequences of a reduced expression of *Foxe3 *[38]. Two amino acid substitutions in the DNA-binding domain of *Foxe3 *are responsible for the dysgenic lens mouse mutant phenotype [37,38], which includes loss of lens epithelium in a small cataractic lens [53]. *Foxe3*-null mice have small lenses as a result of a diminished proliferation in the lens epithelium. Fibre cells do not differentiate properly, showing irregular shapes and persisting nuclei. The lens eventually develops several vacuoles and cataracts [54]. *Foxe3 *down-regulation in lens fibres is crucial for proper differentiation of these cells. Persistent expression of this gene in the posterior region of the lens leads to a partial epithelialisation of fibre cells, with severe consequences for lens function [55]. Mice in which *Foxe3 *is ectopically expressed in fibre cells exhibit lenses with numerous vacuoles and cavities, irregular shape of lens fibre and disorganised cytoskeleton [55].

These anomalies in mice mutant for *Foxe3 *resemble in many respects the abnormal phenotype seen in the mole, and are in accordance with the loss of *FOXE3 *expression seen in many lens epithelial cells and the ectopic expression observed in lens fibres. Consistent with the known roles of Foxe3 in mice, the mole lens has a low proliferation rate and all proliferating lens fibres expressed *FOXE3*. Furthermore, no *FOXE3 *expression was detected in the mole lens at any postnatal stage, with vacuoles appearing just when this gene is down-regulated.

In contrast to *FOXE3*, the expression of *PAX6 *continues in all lens cells throughout the whole life of the mole, and hence, unlike in mice, patterns of *FOXE3 *expression do not parallel those of *PAX6*. Accordingly, we propose that PAX6 may not directly regulate *FOXE3 *in the Iberian mole.

### PAX6 does not repress β-crystallin gene expression in the Iberian mole

Studies on crystallin regulation have been mainly carried out using mice and chickens as animal models. However, there are remarkable differences regarding the crystallin gene expression profile between both species. For example, the chicken lens does not express the γA-F crystallin cluster and contains two taxon-specific δ1 and δ2-crystallins. Moreover, different studies have shown that some regulatory regions of the mouse and chicken crystallins are not highly evolutionary conserved [16]. Nevertheless, this does not seem to be the case for β-crystallins, as the chicken βB1-crystallin promoter is fully functional in transgenic mice [56]. In both rodents and chickens, genes encoding β-crystallins are first expressed in the elongating primary fibre cells and its expression is maintained in this cell type until adulthood. β-crystallins are never found in the lens epithelium, being specific markers of differentiated lens fibres [56-58].

PAX6 represses the chicken βB1-crystallin by displacing PROX1 and MAF transcription factors (which act as positive regulators) from the βB1-crystallin promoter [36]. Cui et al [36] proposed that PAX6 has a direct inhibitory role on the promoter of the gene encoding βB1-crystallin, such that this crystallin is not expressed in PAX6-positive epithelial cells, but in PAX6-negative lens fibres. In the mouse, endogenous βB1-crystallin levels are repressed in lenses overexpressing *Pax6 *in lens fibre cells, suggesting that the βB1-crystallin gene is also negatively regulated by Pax6 in mammals [28]. Nevertheless, our results in the Iberian mole suggest that the direct role of murine Pax6 in controlling β-crystallin expression is not conserved. Newborn moles at stage s9 showed high β-crystallin levels in both epithelial cells and lens fibres, a situation which is maintained until adulthood, even though all mole lens cells also express *PAX6*. It would be worthwhile to perform a proteomic analysis of lens crystallins in the Iberian mole to determine which β-crystallins are present.

Similarly, the results described in this paper in relation to c-MAF and PROX1, which are reported to cooperate in the activation of β-crystallin gene expression [28,36,59], also suggest evolutionary divergence of genetic pathways in the mole. PROX1/Prox1 is involved in the progression of terminal fibre differentiation, because lens cells fail to polarise and elongate properly in *Prox1*^-/- ^mice [60]. Its function seems to be controlled by changes in its sub-cellular distribution during development [40]. The PROX1 protein is predominately cytoplasmic in the lens placode, the lens epithelium and germinative zone throughout development of chickens, rodents and humans. However, this protein is translocated to the nucleus during fibre cell differentiation [40]. Once in the nucleus, mouse Prox1 can bind to CBP/p300, a transcription cofactor, and activate the βB2-crystallin promoter by cooperating with the c-Maf protein [59]. Similarly, both Prox1 and c-Maf work synergistically in the activation of the chicken βB1-crystallin [36]. In the present study, we have detected by immunohistochemistry that the mole PROX1 protein is initially cytoplasmic in the cells that constitute the lens placode, and becomes nuclear at the lens vesicle stage. However, mole PROX1 distribution remains primarily nuclear in both the epithelium and lens fibres until adulthood, and can be detected in the cytoplasm of the elongated fibre cells from stage s6.

*c-MAF *is only weakly expressed in epithelial cells in the Iberian mole. This fact could explain the absence of β-crystallins in the mole lens epithelium during almost the whole prenatal development, because *c-Maf *up-regulation in lens fibres has been described as essential for β-crystallin expression in mice [41] and chickens [36]. However, when β-crystallins are highly expressed in the lens epithelium of infant moles, most of the epithelial cell nuclei appear c-MAF-negative, suggesting that c-MAF is dispensable for β-crystallin expression in the mole.

Taken together, our results clearly suggest that there is no evolutionarily conserved relationship between PAX6, PROX1 and c-MAF in relation to β-crystallin expression in the Iberian mole. A possible explanation for this could be the presence of specific mutations in promoters of mole crystallin genes, something that needs to be investigated in the near future. Another possibility is that all members of the complex network of transcription factors involved in the regulation of crystallin production are not yet completely elucidated, and that other unknown factor(s), which may be lacking or mutated in the Iberian mole, is (are) also necessary in this process.

Owing to the lack of molecular approaches on eye development in true moles, it is not possible for us to establish whether the anomalies observed in the Iberian mole are more severe than would be observed in mole species with open eyelids. Nevertheless, as stated above, we do not believe that Iberian moles are totally blind and regard it as unlikely that the European mole, which is evolutionarily very close to the Iberian mole but has open eyes, shows great visual acuity. The eyes of European moles are less sensitive to light, by several orders of magnitude, than those of humans [45], and one of their primary functions is probably for maintenance of biological rhythms – even with closed eyelids, the Iberian mole may therefore, be able to detect enough light to fulfil this function. Morphological studies suggest a degree of lens abnormality in European moles that is superficially as severe as that found in the Iberian mole [9,64]. This would suggest that the defective (compared with mouse) eye phenotype described in this paper is perhaps highly conserved in the Old World moles of genus *Talpa*. To understand how the adaptation to dark habitats has modified the visual system of these fossorial mammals, it would be necessary to examine several taxa in a phylogenetic context. However, the significant difficulties associated with obtaining fresh embryonic material from such evasive wild animals are likely to make this fascinating challenge a more long-term project.

Further studies on other animal models of eye degeneration, similar to that reported in the present paper, may shed more light on the molecular pathways that control the mammalian lens development and function. Furthermore, research on these natural systems can provide useful information about the evolutionary mechanisms that lead to the atrophy of an organ as important as the eye, and how natural selection worked at the molecular level thus inducing morphogenetic and functional changes in evolving organisms.

## Conclusion

Fossorial animals represent natural models of dysgenic eyes in which molecular pathways involved in eye development have been modified by natural selection. This paper reports for the first time a molecular study of the entire process of lens development, from the establishment of the lens placode to adulthood, in a species of subterranean mammals, the Iberian mole. Our results show that there are primary developmental defects in the lens of this insectivore: lens fibre differentiation is not completed and there is loss of the lens epithelial phenotype, with most epithelial cells undergoing apoptosis. As a result, the adult lens is composed of a disrupted lens epithelium and a disorganised mass of immature and nucleated fibre cells. An impaired regulation of both *PAX6 *and *FOXE3 *seems to be the cause of this abnormal phenotype. Furthermore, our data clearly show that the role of PAX6, c-MAF and PROX1 in relation to the control of genes encoding β-crystallins is not conserved in the Iberian mole. The presence of crystallins in the undeveloped mole lens may explain the fact that there are no acute cataracts, and suggests that the lens of the Iberian mole is probably partially functional. Studying different talpid species would clarify whether the abnormalities observed during lens development in the Iberian mole are specifically associated with the permanent closure of the eyes, or are conserved and shared in moles with open eyes. Furthermore, more research on these naturally modified visual systems would contribute current knowledge about human eye degeneration and disease.

## Methods

### Material analysed

A total of 64 embryos, foetuses, infant and adult moles of the species *Talpa occidentalis *were analysed in this study. As moles do not breed in captivity, all samples were obtained from wild animals captured in poplar groves in the localities of Santa Fe and Chauchina (in the cultivated area around Granada, in southern Spain), as described in Barrionuevo et al [61].

Embryos and foetuses were obtained from pregnant females. Pregnancy was detected by abdominal palpation of females and the developmental stage of the embryos according to the size of the uterine swellings. Infants were collected directly from their nests by radio-tracking lactating females (recognisable by the size and colour of their teats). After dissection, precise developmental staging was carried out based on crown-rump length, body mass values and on the morphology of major external structures, according to Barrionuevo et al [62] and Carmona et al [63].

Captures were performed under annual permission granted by the Andalussian Environmental Council, and animal handling followed the guidelines and approval of the University of Granada's 'Ethical Committee for Animal Experimentation'.

Four litters of CBA/Ca mouse embryos (E12, 15, 16 and 18) and several adult mice, provided by the Medical Research Facility of the University of Aberdeen, were used as a positive control.

### Immunohistochemistry

Embryonic and s9 newborn infant heads and eyes of the rest of the stages were dissected out and fixed in paraformaldehyde solution (4%) overnight at 4°C. Samples were then washed with phosphate buffered saline (PBS), dehydrated, embedded in paraffin wax and sectioned in a microtome, following standard procedures.

For gene expression analyses, sections were de-waxed using histoclear, rehydrated and washed with PBS. Slides were placed in 0.01 M citrate buffer pH6 and boiled for 20 minutes in a microwave for antigen retrieval. Samples were subsequently washed with Tris-buffered saline (TBS) (12.5 mM Tris, 0.9% NaCl, pH7.6), blocked with PBS, 0.3% bovine serum, 4% normal serum (according to the species in which the secondary antibody was developed) and incubated overnight in specific primary antibodies at 4°C.

3,3'-Diaminobenzidine tetrahydrochloride (DAB) immunostaining was performed using secondary biotinylated-antibodies, previous incubation with 3% hydrogen peroxide in PBS for 20 minutes to block endogenous peroxidise activity. After washing with TBS, sections were exposed to ABC-streptavidin horseradish peroxidase complex (Vector Laboratories, Peterborough, UK) for 25 minutes at room temperature, washed again in TBS and then incubated in 0.83 mg/ml DAB, 20 mM Tris, pH 7.6, 1/6000 hydrogen peroxide in the dark for up to 45 minutes at room temperature. Subsequent haematoxylin staining was carried out in DAB-stained samples.

Secondary fluorochrome-conjugated antibodies were used for immunofluorescence assays and photomicrographs taken using a red/green single bandpass fluorescence mirror unit. Table 1 summarises the antibodies used in this study.

**Table 1:** Primary antibodies used in this study

**Gene product**^1^	**Antibody source**	**Working dilution**	**References**
PAX6	Mouse monoclonal, raised against chicken protein	1/400	Developmental Studies Hybridoma Bank, University of Iowa, US
PROX1	Rabbit polyclonal, raised against human protein	1/250	Sigma (P7124)
c-MAF	Goat polyclonal, raised against human protein	1/200	Santa Cruz (sc-10017)
FOXE3	Goat polyclonal, raised against mouse protein	1/200	Santa Cruz (sc-48162)
β-crystallin	Rabbit polyclonal, raised against human protein	1/100	Santa Cruz (sc-22745)
PH3	Rabbit polyclonal raised against human protein	1/100	Upstate (06–570)

^1 ^Gene product names: PAX6, paired box gene 6; PROX1, prospero-related homeobox 1; c-MAF, v-maf musculo-aponeurotic fibrosarcoma (avian) oncogene homolog; FOXE3, forkhead box E3; PH3, phosphorylated histone H3.

### TUNEL labelling

To identify apoptotic cells, *in situ *TUNEL reaction was carried out using the 'In Situ Cell Death Detection Kit, Fluorescein' (Roche, Cat. No. 11684795910).

### TEM

Samples for TEM were processed by the Histology and Electron Microscopy Facility of the Institute of Medical Sciences, University of Aberdeen. Electron micrographs were obtained using a Philips CM10 TEM microscope.

## Abbreviations

DAB: 3,3'-diaminobenzidine tetrahydrochloride; dpc: days post-coitum; PBS: phosphate buffered saline; TBS: Tris-buffered saline; TEM: transmission electron microscopy; TUNEL: terminal deoxynucleotidyl transferase (TdT)-mediated dUTP nick-end labelling.

## Authors' contributions

FDC performed all experiments. JMC and FDC jointly planned the project and wrote the manuscript. RJ was responsible for the preparation of the samples as well as for reviewing the manuscript.
